# Prognostic value of inflammation biomarkers for 30-day mortality in critically ill patients with stroke

**DOI:** 10.3389/fneur.2023.1110347

**Published:** 2023-02-06

**Authors:** Jun Zhao, Jinli Feng, Qian Ma, Chunlin Li, Feng Qiu

**Affiliations:** ^1^Senior Department of Neurology, The First Medical Center of PLA General Hospital, Beijing, China; ^2^Department of Health Medicine, The Eighth Medical Center of PLA General Hospital, Beijing, China

**Keywords:** inflammation, biomarkers, 30-day mortality, ischemic stroke, hemorrhagic stroke

## Abstract

**Objective:**

To explore the values of neutrophil to lymphocyte ratio (NLR), platelet to lymphocyte ratio (PLR), neutrophil to albumin ratio (NAR), prognostic nutritional index (PNI), systemic immune inflammatory index (SII) and red cell distribution width to albumin ratio (RA) for evaluating the risk of 30-day mortality of ischemic stroke or hemorrhagic stroke patients.

**Methods:**

In this cohort study, the data of 1,601 patients diagnosed with stroke were extracted from the Medical Information Mart for Intensive Care III (MIMIC-III) database. Among them, 908 were hemorrhagic stroke patients and 693 were ischemic stroke patients. Demographic and clinical variables of patients were collected. Univariate and multivariable Cox regression were performed to evaluate the predictive values of NLR, PLR, SII, NAR, RA, and PNI for 30-day mortality in hemorrhagic stroke or ischemic stroke patients. The receiver operator characteristic (ROC) curves were plotted to assess the predictive values of NLR, NAR, and RA for 30-day mortality of hemorrhagic stroke patients.

**Results:**

At the end of follow-up, 226 hemorrhagic stroke patients and 216 ischemic stroke patients died. The elevated NLR level was associated with increased risk of 30-day mortality in hemorrhagic stroke [hazard ratio (HR) = 1.17, 95% confidence interval (CI): 1.06–1.29]. The increased NAR level was associated with elevated risk of 30-day mortality in hemorrhagic stroke (HR = 1.16, 95% CI: 1.02–1.30). The high RA level was linked with increased risk of 30-day mortality (HR = 1.44, 95% CI: 1.23–1.69). No significant correlation was observed in these inflammation biomarkers with the risk of 30-day mortality in ischemic stroke patients. The area under the curves (AUCs) of NLR, RA, and NAR for evaluating the risk of 30-day mortality of hemorrhagic stroke patients were 0.552 (95% CI: 0.503–0.601), 0.644 (95% CI: 0.590–0.699) and 0.541 (95% CI: 0.490–0.592).

**Conclusion:**

NLR, NAR, and RA were potential prognostic biomarkers for predicting 30-day mortality of hemorrhagic stroke patients, which might provide clinicians an easy and cheap way to quickly identify patients with high risk of mortality.

## Introduction

Stroke is a serious disease affecting a quarter of people during their lifetime with high risk of death and disability (1). Stroke has two main subtypes (ischemic stroke and hemorrhagic stroke), and they have distinct clinical and epidemiological characteristics (2). Ischemic stroke and hemorrhagic stroke are accounted for ~85 and 15% of all stroke cases, respectively (3). Ischemic stroke is caused by the reduction or interruption of blood flow to the brain while hemorrhagic stroke is due to the bleeding in or around the brain (4). Ischemic stroke is the major cause of disability and second cause of deaths globally with a mortality rate of 15% at 90 days (5, 6). As for hemorrhagic strokes, the mortality rate is 25–30% in high-income countries and 30%-48% in low- to middle-income countries (7). Given the prognosis of stroke patients, more reliable biomarkers were essential to help improve the outcomes of these patients.

Numerous studies have demonstrated that neuroinflammatory response plays an essential role in the pathophysiology of ischemic stroke (8, 9). Inflammation associated biomarkers such as monocyte and plateletcrit were reported to be associated with the development of cerebrovascular events including acute ischemic stroke (10, 11). Recently, neutrophil to lymphocyte ratio (NLR), platelet to lymphocyte ratio (PLR), neutrophil-albumin ratio (NAR), prognostic nutritional index (PNI), systemic immune inflammatory index (SII) and red cell distribution width (RDW) to albumin ratio (RA) have been reported as potential novel biomarkers of baseline inflammatory process and they were identified to be associated with the mortality of stroke (12–17). These studies mainly explored the associations between these inflammation biomarkers and all stroke patients or ischemic stroke patients. They did not compare the differences of these inflammation biomarkers with the mortality of different subtypes of stroke patients. Whether there were differences in the prognostic values of these inflammation biomarkers between ischemic stroke and hemorrhagic stroke was unclear. Which inflammation biomarker was more clearly related to the prognosis of ischemic stroke or hemorrhagic stroke still needs investigation.

In the present study, we hypothesized that ischemic stroke and hemorrhagic stroke might have different prognostic inflammation biomarkers. We planned to explore the prognostic values of NLR, PLR, NAR, PNI, SII, and RA for 30-day mortality of ischemic stroke or hemorrhagic stroke patients based on the data from the Medical Information Mart for Intensive Care III (MIMIC-III) to verify our hypothesis.

## Methods

### Study population

In the current cohort study, the data of 3,534 patients diagnosed with stroke were extracted from MIMIC-III database. MIMIC-III is a large, free database involving in de-identified health-related data of over 40,000 patients who stayed in intensive care unit (ICU) of the Beth Israel Deaconess Medical Center (Boston, USA) between 2001 and 2012 (18). The data analyzed using the first measurement data within 24 h after admitting to ICU. Patients who aged < 18 years, and those who stayed in ICU < 24 h were excluded. Those who had no data on SII, NAR, systolic blood pressure (SBP), international normalized ratio (INR), Glasgow coma scale (GCS), or Elixhauser comorbidity index (ECI), and patients with abnormal follow-up time were also excluded. Finally, 1,601 patients were included. Among them, 908 were hemorrhagic stroke patients and 693 were ischemic stroke patients. The project was approved by the Institutional Review Boards of Beth Israel Deaconess Medical Center (Boston, MA) and the Massachusetts Institute of Technology (Cambridge, MA). Requirement for individual patient consent was waived because the project did not impact clinical care and all protected health information was deidentified. As the samples were not from The Eighth Medical Center of PLA General Hospital, and this study was exempt from our Institutional Review Board approval.

### Main variables

Main variables analyzed in our study included NLR, PLR, NAP, PNI, SII and RA. NLR (neutrophil to lymphocyte ratio) = neutrophil count/lymphocytes count. PLR (platelet to lymphocyte ratio) = platelet count/lymphocytes count. NAR (neutrophil to albumin ratio) = neutrophil count/albumin. PNI (prognostic nutritional index) = 10 × albumin (g/dL) + 5 × lymphocytes count (10^9^/L). SII (systemic immune inflammatory index) = PLT × neutrophil/lymphocyte. RA (RDW to albumin ratio) = RDW/albumin (g/dL).

### Potential covariables and definition

Potential covariables analyzed in this study included demographic variables including age (years), gender (female or male), marital status (married, unmarried or unknown), and race [White, or others (Asian, Black, Hispanic or Latino, Unknown)], and clinical variables including respiratory rate (beat/min), SBP (mmHg), diastolic blood pressure (DBP, mmHg), blood oxygen saturation (SpO_2_), red blood count (RBC, m/μL), INR, hemoglobin (g/dL), hematocrit (%), creatinine (mg/dL), blood urea nitrogen (BUN, mg/dL), fasting blood-glucose (mg/dL), sodium, potassium, chloride, bicarbonate (mEq/L), ECI score, GCS Score, acute kidney failure (AKI, yes or no), infection diseases and treatments.

Infectious diseases was identided from MIMIC-III database based on the ICD-9 code with the first three digits of 001–009, 010–018, 020–027, 030–042, 045–049, 050–059, 060–066, 070–079, 080–088, 090–099, 100–104, 110–118, 120–129, 130–136, and 137–139. Treatments of ischemic stroke included intravenous tissue plasminogen activator (IV-tPA) (ICD-9 procedure code 9910 and 3604), endovascular treatment (ICD-9 procedure code 3974), and the ICD for stent in the procedure (0045, 0046, 0047, and 0048). The main treatments for hemorrhagic stroke were surgery including craniotomy (ICD-9 procedure code: 0120–0129), and minimally invasive surgery (ICD-9 procedure code 0221, 0222, 0139, 0101, 0102, and 0109).

### Outcome variable

The 30-day mortality of patients was regarded as outcome in our study. The median follow-up was 30 (21.38, 30.00) days. The follow-up was ended when patients died within 30 days. The outcome was obtained through in-hospital observations or through the Social Security Number of patients. At the end of follow-up, 226 hemorrhagic stroke patients and 216 ischemic stroke patients died.

### Statistical analysis

Normally distributed measurement data were described as mean and standard deviation (Mean ± SD), while non-normally distributed measurement data were shown as median and quartile spacing [M (Q_1_, Q_3_)]. Mann-whitney U rank-sum test was applied for comparison between groups. Enumeration data were expressed as n (%), and χ^2^ test was used for comparisons between groups. Univariate cox models were established for 30-day mortality and hazards ratio (HR) and 95% confidence interval (CI) were standardized with *P* < 0.05 as potential covariables. Univariate and multivariable cox regression were performed to evaluate the prognostic values of NLR, PLR, SII, NAR, RA, and PNI for 30-day mortality of hemorrhagic stroke or ischemic stroke patients. To evaluate the associations between NLR, PLR, SII, NAR, RA, or PNI and 30-day mortality in hemorrhagic stroke patients, confounding factors including age, marital status, respiratory rate, hemoglobin, hematocrit, BUN, fasting blood-glucose, chloride, ECI and AKI were adjusted in the multivariable cox regression model. To assess the associations between NLR, PLR, SII, NAR, RA, or PNI and 30-day mortality in ischemic stroke patients, age, marital status, race, creatinine, BUN, bicarbonate, potassium, ECI, GCS, and AKI were adjusted in the multivariable cox regression model. The receiver operator characteristic (ROC) curves were plotted to evaluate the diagnostic values of NLR, NAR, and RA for 30-day mortality of hemorrhagic stroke. R Studio Version 4.0.3 (2020-10-10) and SAS 9.4 (SAS Institute Inc., Cary, USA) were applied for data analysis.

## Results

### The baseline characteristics of patients with hemorrhagic stroke or ischemic stroke

**Figure 1:**
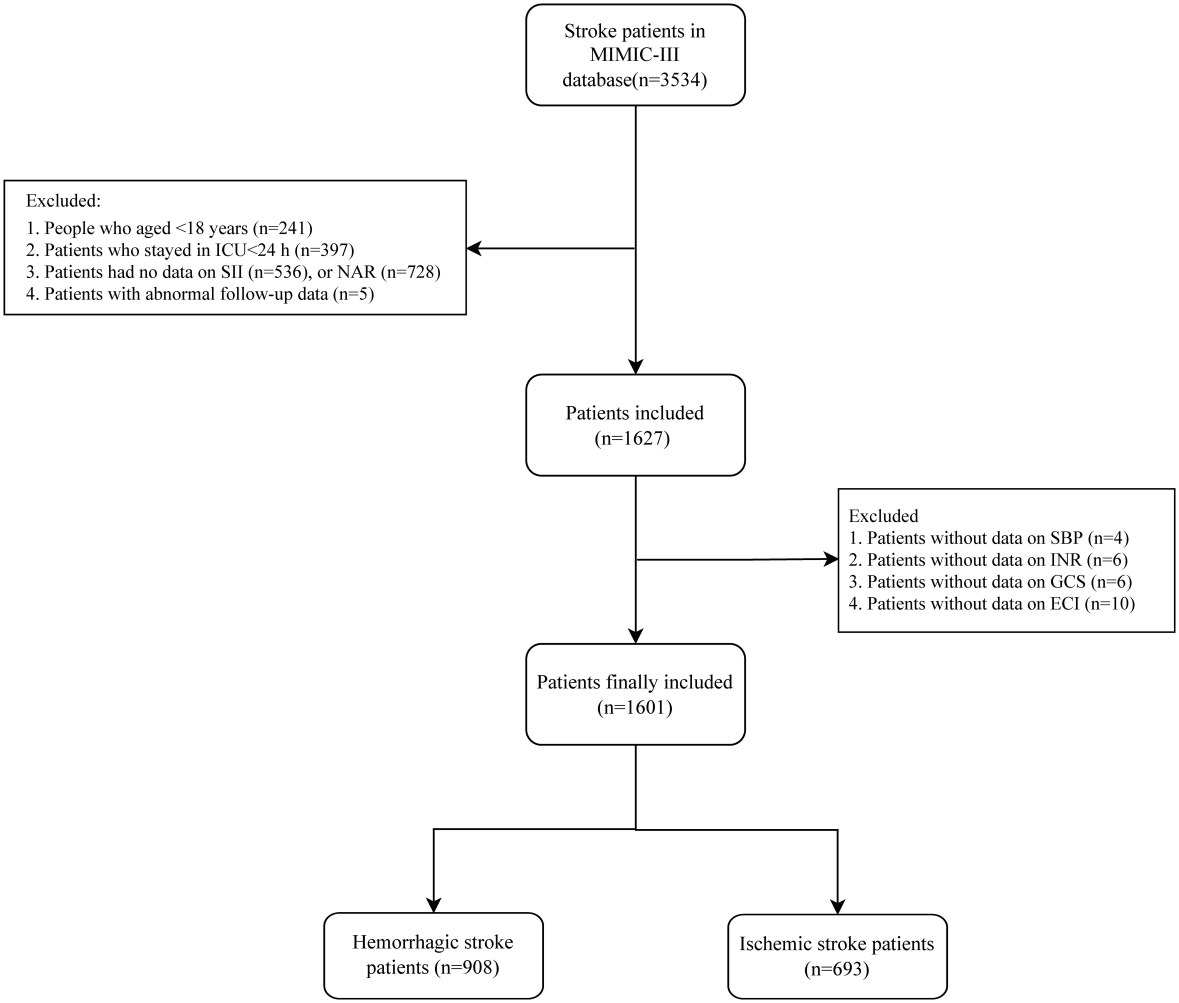
The screen process of the participants in the current study.

In total, 3,534 stroke patients were found in MIMIC-III database, among them, 241 people who aged < 18 years and 397 patients who stayed in ICU < 24 h were excluded. Five hundred and thirty-six patients had no data on SII and 728 patients had no data on NAR, and they were excluded. Five patients with abnormal follow-up data (the day admitted to ICU was after the death day) were excluded. Patients without data on SBP (*n* = 4), INR (*n* = 6), GCS (*n* = 6) and ECI (*n* = 10) were not included. Finally, 1,601 stroke patients were involved in with 908 hemorrhagic stroke patients and 693 ischemic stroke patients. The screen process was shown in Figure 1.

As observed in Table 1, the median NLR (8.39 vs. 7.06) and RA (4.58 vs. 3.97) in the death group were higher than the survival group in hemorrhagic stroke patients. The median PNI in the death group was lower than the survival group in hemorrhagic stroke patients (39.90 vs. 43.17). The median NLR (8.17 vs. 6.82), SII (1.90 vs. 1.58), NAR (2.93 vs. 2.51) and RA (4.52 vs. 4.26) in the death group were higher than the survival group in patients with ischemic stroke. The median PNI in the death group was lower than the survival group in patients with ischemic stroke (38.64 vs. 41.11).

**Table 1:** Baseline characteristics between patients survived and died within 30 days with ischemic stroke or hemorrhagic stroke.

	**Hemorrhagic stroke**			**Ischemic stroke**		
**Variables**	**Survival group (*****n*** = **682)**	**Death group (*****n*** = **226)**	***P***	**Survival (*****n*** = **477)**	**Death group (*****n*** = **216)**	***P***
Age, years M (Q_1_, Q_3_)	62.79 (51.52, 74.13)	74.44 (59.17, 81.98)	< 0.001	68.02 (55.69, 78.13)	77.26 (66.44, 84.51)	< 0.001
Gender, *n* (%)			0.733			0.402
Female	314 (46.04)	107 (47.35)		242 (50.73)	117 (54.17)	
Male	368 (53.96)	119 (52.65)		235 (49.27)	99 (45.83)	
Marital status, n (%)			0.001			0.024
Married	351 (51.47)	106 (46.90)		227 (47.59)	90 (41.67)	
Unmarried	289 (42.38)	89 (39.38)		222 (46.54)	101 (46.76)	
Unknown	42 (6.16)	31 (13.72)		28 (5.87)	25 (11.57)	
Race, *n* (%)			0.179			0.037
White	482 (70.67)	149 (65.93)		325 (68.13)	164 (75.93)	
Others*	200 (29.33)	77 (34.07)		152 (31.87)	52 (24.07)	
Respiratory rate, Mean ± SD	17.65 ± 4.49	18.56 ± 5.76	0.031	18.00 (14.00, 22.00)	19.00 (16.00, 24.00)	0.025
SBP, mmHg, Mean ± SD	140.26 ± 25.94	138.92 ± 28.27	0.512	136.16 ± 27.82	135.00 ± 31.68	0.646
DBP, mmHg, Mean ± SD	72.17 ± 17.12	70.16 ± 19.44	0.165	69.39 ± 18.28	69.40 ± 20.70	0.994
SpO_2_, Mean ± SD	97.93 ± 4.92	97.63 ± 5.47	0.476	97.50 ± 4.80	97.13 ± 4.00	0.286
RBC, m/ul, Mean ± SD	4.33 ± 0.64	4.12 ± 0.82	< 0.001	4.07 ± 0.75	3.99 ± 0.72	0.214
INR, M (Q_1_, Q_3_)	1.10 (1.00, 1.20)	1.20 (1.10, 1.60)	< 0.001	1.20 (1.10, 1.30)	1.20 (1.10, 1.40)	< 0.001
Hemoglobin, g/dL, Mean ± SD	13.13 ± 1.89	12.54 ± 2.31	< 0.001	12.22 ± 2.30	12.01 ± 2.16	0.258
Hematocrit, percent, Mean ± SD	38.38 ± 5.30	36.95 ± 6.52	0.003	36.22 ± 6.35	35.83 ± 5.98	0.451
Creatinine, mg/dl, M (Q_1_, Q_3_)	0.90 (0.70, 1.10)	1.00 (0.80, 1.30)	< 0.001	1.00 (0.80, 1.40)	1.20 (0.90, 1.70)	< 0.001
BUN, mg/dl, M (Q_1_, Q_3_)	16.00 (13.00, 22.00)	20.00 (15.00, 28.00)	< 0.001	19.00 (14.00, 29.00)	25.00 (16.00, 41.00)	< 0.001
Fasting blood-glucose, mg/dl, M (Q_1_, Q_3_)	138.00 (116.00, 169.00)	154.50 (126.00, 213.00)	< 0.001	127.00 (108.00, 167.00)	136.00 (111.00, 170.50)	0.044
Bicarbonate, mEq/L, Mean ± SD	24.48 ± 3.40	24.05 ± 4.04	0.152	24.38 ± 4.36	23.35 ± 4.64	0.005
Sodium, Mean ± SD	138.96 ± 4.14	138.23 ± 5.05	0.049	139.25 ± 4.60	138.60 ± 4.74	0.085
Potassium, Mean ± SD	4.07 ± 0.70	4.13 ± 0.85	0.343	4.19 ± 0.80	4.40 ± 0.92	0.005
Chloride, Mean ± SD	103.13 ± 4.71	101.93 ± 5.84	0.005	103.79 ± 5.90	102.93 ± 5.82	0.075
Bicarbonate, mEq/L, Mean ± SD	24.48 ± 3.40	24.05 ± 4.04	0.152	24.38 ± 4.36	23.35 ± 4.64	0.005
Infection diseases, *n* (%)			0.106			0.446
No	472 (98.95)	210 (97.22)		555 (81.38)	189 (83.63)	
Yes	5 (1.05)	6 (2.78)		127 (18.62)	37 (16.37)	
IV-tPA, *n* (%)			0.130	–	–	–
No	411 (86.16)	195 (90.28)		—-	–	
Yes	66 (13.84)	21 (9.72)		–	–	
Endovascular treatment, *n* (%)			0.586	–	–	–
No	455 (95.39)	208 (96.30)		–	–	
Yes	22 (4.61)	8 (3.70)		–	–	
Craniotomy, *n* (%)	–	–	–			0.137
No	–	–		644 (94.43)	219 (96.90)	
Yes	–	–		38 (5.57)	7 (3.10)	
Minimally invasive surgery, *n* (%)	–	–	–			
No	–	–		633 (92.82)	208 (92.04)	
Yes	–			49 (7.18)	18 (7.96)	
ECI, M (Q_1_, Q_3_)	6.00 (0.00, 13.00)	9.00 (0.00, 16.00)	0.019	8.00 (4.00, 16.00)	12.00 (6.00, 18.50)	< 0.001
GCS score, M (Q_1_, Q_3_)	14.00 (11.00, 15.00)	14.00 (7.00, 15.00)	0.304	14.00 (11.00, 15.00)	14.00 (9.00, 15.00)	0.032
AKI, *n* (%)			< 0.001			< 0.001
No	353 (51.76)	80 (35.40)		191 (40.04)	51 (23.61)	
Yes	329 (48.24)	146 (64.60)		286 (59.96)	165 (76.39)	
NLR, M (Q_1_, Q_3_)	7.06 (4.01, 11.53)	8.39 (4.41, 13.17)	0.020	6.82 (3.57, 11.54)	8.17 (4.80, 14.31)	0.002
PLR, M (Q_1_, Q_3_)	184.71 (124.64, 268.00)	188.28 (104.73, 316.82)	0.850	182.60 (117.94, 306.12)	211.89 (134.90, 309.61)	0.295
SII, M (Q_1_, Q_3_)	1,712.54 (888.13, 2,750.82)	1,701.81 (676.02, 2,992.99)	0.861	1,577.03 (814.29, 2,812.50)	1,900.55 (891.06, 3,461.58)	0.028
NAR, M (Q_1_, Q_3_)	2.45 (1.66, 3.33)	2.62 (1.72, 4.03)	0.062	2.51 (1.58, 3.77)	2.93 (1.94, 4.18)	< 0.001
PNI, M (Q_1_, Q_3_)	43.17 (38.39, 48.33)	39.90 (34.10, 46.40)	< 0.001	41.11 (34.94, 46.45)	38.64 (33.21, 43.19)	< 0.001
RA, Mean ± SD	3.97 ± 0.87	4.58 ± 1.31	< 0.001	4.26 (3.66, 5.22)	4.52 (3.90, 5.42)	0.003
Lymphocytes, M (Q_1_, Q_3_)	11.80 (7.60, 18.40)	10.00 (6.00, 16.80)	0.003	12.00 (7.20, 19.80)	10.00 (6.00, 15.80)	0.002
Neutrophil,%, Mean ± SD	79.02 ± 13.12	78.97 ± 16.04	0.964	77.07 ± 14.47	79.40 ± 13.63	0.046
RDW, percent, Mean ± SD	13.96 ± 1.46	14.85 ± 2.14	< 0.001	14.69 ± 2.05	14.89 ± 1.79	0.189
PLT, K/uL, M (Q_1_, Q_3_)	241.00 (184.00, 295.00)	215.00 (142.00, 287.00)	< 0.001	241.00 (175.00, 309.00)	236.00 (162.50, 300.50)	0.420
Albumin,%, Mean ± SD	3.62 ± 0.56	3.40 ± 0.65	< 0.001	3.34 ± 0.67	3.19 ± 0.63	0.007

SBP, systolic blood pressure; DBP, diastolic blood pressure; SpO_2_, blood oxygen saturation; RBC, red blood count; INR, international normalized ratio; BUN, blood urea nitrogen; GCS, Glasgow coma scale; IV-tPA, intravenous tissue plasminogen activator; ECI, Elixhauser comorbidity index; AKI, acute kidney failure; NLR, neutrophil to lymphocyte ratio; PLR, platelet to lymphocyte ratio; SII, systemic immune inflammatory index; NAR, neutrophil to albumin ratio; PNI, prognostic nutritional index; RA, red cell distribution width to albumin ratio.

Others^*^: Asian, Black, Hispanic or Latino, Unknown.

### Potential covariables associated with 30-day mortality in hemorrhagic or ischemic stroke patients

Potential covariables with statistical difference in Table 1 was involved in univariate cox analysis. The results depicted that age (HR = 1.01, 95% CI: 1.00–1.01), marital status, respiratory rate (HR = 1.03, 95% CI: 1.00–1.06), RBC (HR = 0.60, 95% CI: 0.38–0.95), hemoglobin (HR = 0.74, 95% CI: 0.58–0.95), hematocrit (HR = 0.96, 95% CI: 0.94–0.98), BUN (HR = 1.01, 95% CI: 1.01–1.01), fasting blood–glucose (HR = 1.00, 95% CI: 1.00–1.01), chloride (HR = 0.96, 95% CI: 0.94–0.99), ECI (HR = 1.02, 95% CI: 1.00–1.03) and AKI (HR = 1.74, 95% CI: 1.32–2.29) were covariables that might be associated with 30–day mortality in hemorrhagic stroke patients (Table 2). Age (HR = 1.00, 95% CI: 1.00–1.01), marital status, race (HR = 1.39, 95% CI: 1.02–1.91), creatinine (HR = 1.08, 95% CI: 1.00–1.16), BUN (HR = 1.01, 95% CI: 1.00–1.02), bicarbonate (HR = 0.96, 95% CI: 0.93–0.99), potassium (HR = 1.26, 95% CI: 1.09–1.45), ECI (HR = 1.03, 95% CI: 1.01–1.04), GCS (HR = 0.93, 95% CI: 0.90–0.97) and AKI (HR = 1.95, 95% CI: 1.42–2.67) were covariables that might be associated with 30-day mortality in ischemic stroke patients (Table 3).

**Table 2:** Potential covariables associated with 30-day mortality in hemorrhagic stroke patients.

**Variables**	**HR (95% CI)**	***P***
Age	1.01 (1.00-1.01)	< 0.001
Marital status		
Married	Ref	
Unknown	2.10 (1.41–3.13)	< 0.001
Unmarried	1.01 (0.76–1.33)	0.961
Respiratory rate	1.03 (1.00–1.06)	0.030
RBC	0.60 (0.38–0.95)	0.028
INR	1.04 (1.00–1.09)	0.075
Hemoglobin	0.74 (0.58–0.95)	0.016
Hematocrit	0.96 (0.94–0.98)	< 0.001
Creatinine	1.02 (0.98–1.07)	0.293
BUN	1.01 (1.01–1.01)	< 0.001
Fasting blood-glucose	1.00 (1.00–1.01)	< 0.001
Sodium	0.97 (0.94–1.00)	0.068
Chloride	0.96 (0.94–0.99)	0.002
ECI	1.02 (1.00–1.03)	0.016
AKI	1.74 (1.32–2.29)	< 0.001
Infectious disease		
No	Ref	
Yes	0.86 (0.61–1.23)	0.41
Craniotomy		
No	Ref	
Yes	0.58 (0.27–1.23)	0.153
Minimally invasive surgery		
No	Ref	
Yes	1.12 (0.69–1.81)	0.649

HR, hazard ratio; CI, confidence interval; RBC, red blood count; INR, international normalized ratio; BUN, blood urea nitrogen; ECI, Elixhauser comorbidity index; AKI, acute kidney failure; IV-tPA, intravenous tissue plasminogen activator.

**Table 3:** Potential covariables associated with 30-day mortality in ischemic stroke patients.

**Variables**	**HR (95% CI)**	***P***
Age	1.00 (1.00-1.01)	< 0.001
Marital status		
Married	Ref	
Unknown	1.86 (1.20–2.91)	0.006
Unmarried	1.12 (0.84–1.49)	0.437
Race	1.39 (1.02–1.91)	0.037
Respiratory rate	1.01 (1.00–1.03)	0.076
INR	1.03 (0.94–1.13)	0.480
Creatinine	1.08 (1.00–1.16)	0.036
BUN	1.01 (1.00–1.02)	< 0.001
Fasting blood-glucose	1.00 (1.00–1.00)	0.078
Bicarbonate	0.96 (0.93–0.99)	0.004
Potassium	1.26 (1.09–1.45)	0.001
ECI	1.03 (1.01–1.04)	< 0.001
GCS	0.93 (0.90–0.97)	< 0.001
AKI	1.95 (1.42–2.67)	< 0.001
Infectious disease		
No	Ref	
Yes	2.16 (0.96–4.86)	0.063
IV-tPA		
No	Ref	
Yes	0.74 (0.47–1.15)	0.181
Endovascular treatment		
No	Ref	
Yes	0.86 (0.42–1.73)	0.664

HR, hazard ratio; CI, confidence interval, INR, international normalized ratio, BUN, blood urea nitrogen, ECI, Elixhauser comorbidity index, GCS, Glasgow coma scale, AKI, acute kidney failure, IV-tPA, intravenous tissue plasminogen activator.

### Associations between NLR, PLR, SII, NAR, RA or PNI and 30-day mortality in hemorrhagic stroke or ischemic stroke patients

**Figure 2:**
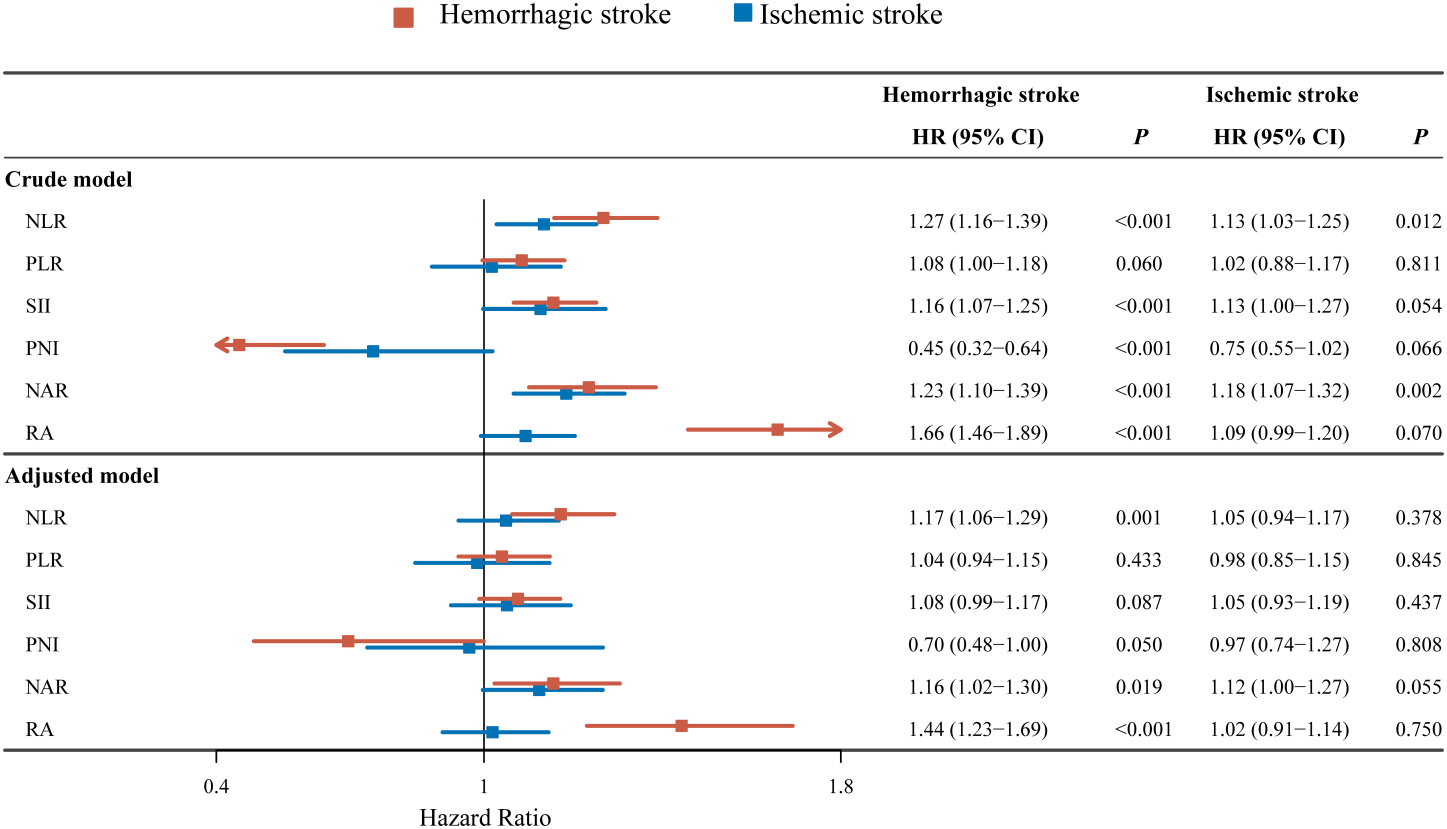
Forest plot showing the associations between NLR, PLR, SII, NAR, RA or PNI and 30-day mortality in hemorrhagic stroke or ischemic stroke patients.

As exhibited in Figure 2, univariate analysis revealed that NLR (HR = 1.27, 95% CI: 1.16–1.39), SII (HR = 1.16, 95% CI: 1.07–1.25), NAR (HR = 0.45, 95% CI: 0.32–0.64), RA (HR = 1.23, 95% CI: 1.10–1.39) or PNI (HR = 1.66, 95% CI: 1.46–1.89) might have associations with 30-day mortality in hemorrhagic stroke patients. After adjusting for confounders including age, marital status, respiratory rate, hemoglobin, hematocrit, BUN, fasting blood-glucose, chloride, ECI and AKI, the elevated NLR level was associated with increased risk of 30-day mortality in hemorrhagic stroke (HR = 1.17, 95% CI: 1.06–1.29). The high level of NAR was associated with elevated risk of 30-day mortality in hemorrhagic stroke (HR = 1.16, 95% CI: 1.02–1.30). The increased level of RA was linked with elevated risk of 30-day mortality (HR = 1.44, 95% CI: 1.23–1.69). The higher level of NLR (HR = 1.13, 95% CI: 1.03–1.25), and NAR (HR = 1.18, 95% CI: 1.07–1.32) might correlate with increased risk of 30-day mortality in ischemic stroke patients. No significant correlation was observed in these inflammation biomarkers with the risk of 30-day mortality in ischemic stroke patients after adjusting for age, marital status, race, creatinine, BUN, bicarbonate, potassium, ECI, GCS and AKI (all *P* > 0.05).

### The predictive values of NLR, NAR, or RA for 30-day mortality in hemorrhagic stroke patients

**Figure 3:**
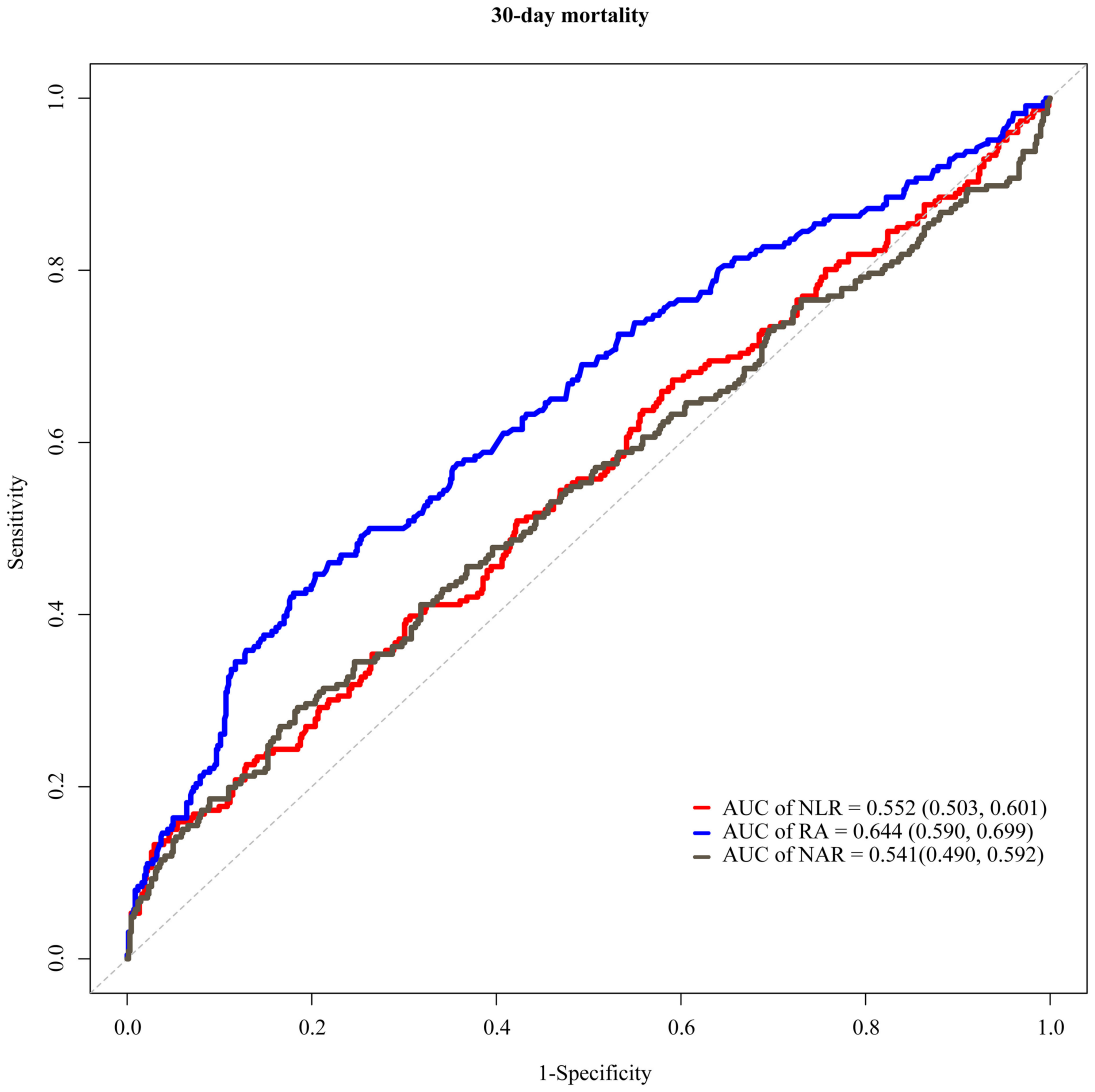
ROC curves presenting the values of NLR, NAR and RA for evaluating the risk of 30-day mortality in hemorrhagic stroke.

The C-indexes of NLR, NAR, and RA for evaluating the 30-day mortality in hemorrhagic stroke patients were 0.54 (95% CI: 0.50–0.58), 0.53 (95% CI: 0.49–0.57), and 0.61 (95% CI: 0.57–0.65), respectively (Table 4). The AUCs were shown in Figure 3. The AUC values of NLR, NAR and RA for evaluating the risk of 30-day mortality for hemorrhagic stroke patients were 0.552 (95% CI: 0.503–0.601), 0.541 (95% CI: 0.490–0.592) and 0.644 (95% CI: 0.590–0.699). Delong test revealed that the AUCs of NLR and NAR were statistically lower than the AUC of RA(*P* < 0.001).

**Table 4:** The C-index of NLR, NAR, and RA for evaluating the risk of 30-day mortality of hemorrhagic stroke patients.

	**C-index (95% CI)**
NLR	0.54 (0.50–0.58)
RA	0.61 (0.57–0.65)
NAR	0.53 (0.49–0.57)

CI, confidence interval; RA, red cell distribution width to albumin ratio; NAR, neutrophil to albumin ratio; NLR, neutrophil to lymphocyte ratio.

## Discussion

In this study, the prognostic values of NLR, PLR, NAR, PNI, SII, and RA for 30-day mortality of ischemic stroke or hemorrhagic stroke patients were investigated based on the data form MIMIC-III database. The results unveiled that high levels of NLR, NAR and RA were linked with increased risk of 30-day mortality in hemorrhagic stroke patients. The AUC values of NLR, NAR, and RA for diagnosing the risk of 30-day mortality in hemorrhagic stroke were 0.552, 0.541, and 0.644. respectively. The findings suggested the values of monitoring the levels of inflammation biomarkers for timely identifying hemorrhagic stroke patients with high risk of mortality within 30 days and provide appropriate interventions to improve their outcomes.

Inflammation is one of the most important pathophysiological mechanisms of stroke and the inflammatory response is activated after stroke, which serves a vital part in secondary brain injury in patients (19). Recently, increasing studies have reported the essential role of immunity in predicting the prognosis and treating patients with acute stroke (20). Immunity is a complex process, and the activation and immunosuppression of different inflammatory cells are induced during the process (21). Neutrophils and lymphocytes are two important inflammatory cells, which were reported to have different roles of in the prognosis after stroke (22, 23). NLR is the ratio of neutrophil to lymphocyte, which can be calculated both from the absolute number of neutrophils and lymphocytes, and from their relative number (24). Previously, a high NLR level was found to associate with poor functional outcomes and increased mortality in patients with spontaneous intracerebral hemorrhage (ICH) (25, 26). These studies provided support to the findings of our study, which depicted that increased NLR was linked with higher risk of 30-day mortality of hemorrhagic stroke patients. NAR is the ratio of neutrophil to albumin, which has become a novel index reflecting systemic inflammation and predicting outcomes of patients in diseases (27). Albumin is an abundant protein in human blood plasma which has osmoregulation, anti-oxidation and anti-inflammation functions (28). A low albumin level was associated with increased mortality risk in hospitalized patients (29). Li et al. found that low serum albumin levels were associated with increased risk of total stroke, ischemic stroke, and ICH (30). In our study, a high level of NAR was correlated to increased risk of 30-day mortality of hemorrhagic stroke patients. RA is another inflammation biomarker derived from the ratio of RDW to albumin, which was reported to be associated with mortality of stroke (17). RDW is a hematologic parameter showing the divergence of red blood cell volume (31). In previous studies, RDW was identified to closely associate with prognosis of cardiovascular events (32, 33). Some other studies revealed that RA might be correlated with hemorrhagic transformation in acute ischemic stroke patients (34). Herein, elevated RA level was associated with higher risk of 30-day mortality of hemorrhagic stroke patients.

The mechanisms underlying the association between NLR, NAR, and RA with 30-day mortality in hemorrhagic stroke patients might be the follows. In hemorrhagic stroke patients, the increased number of neutrophils and decreased number of lymphocytes could induce a cytokine-chemokine storm and, ultimately, lead to more complications (35). Increased neutrophils can release chemical mediators related to increased tissue damage and poor neurological prognosis in stroke patients (36). Lymphocytes were reported to play a brain protective role and the decrease of lymphocytes may lead to deterioration of nerve function (37). Albumin was found to exert an anticoagulant role and inhibitory effect on platelet function by binding antithrombin (38–40), which might aggravate the development of hemorrhagic stroke. In our study, we found that NLR, NAR and RA had potential prognostic values for 30-day mortality in hemorrhagic stroke patients. Previously, ICH score was reported to be a reliable clinical grading scale that allows risk stratification for patients with ICH (41). ICH score includes a basic neurological examination (GCS), a baseline patient characteristic (age), and initial neuroimaging (ICH volume, IVH, infratentorial/supratentorial origin), and compared with ICH score, NLR, NAR and RA are easily available and inexpensive markers that can be routinely detected in clinic. Application of these prognostic biomarkers may help clinicians enhance risk stratification, design individual treatments, and determine follow-up schedules for hemorrhagic stroke patients, which might further improve the outcomes of those patients.

There was evidence indicating that NLR, PLR, or NAR might associate with 30-day mortality of ischemic stroke patients in previous studies (42–44). The mechanisms underlying the findings might be related to the different roles of neutrophils and lymphocytes in the pathophysiologic development of atherosclerosis (45). Neutrophils are found to accumulate in cerebral vessels shortly after stroke and may result in infarctions extension and inhibit microvascular perfusion (46). PNI reflects nutritional status of patients, and previous studies revealed that malnutrition was associated with increased mortality in older Chinese adults with ischemic stroke (47). In our study, no significant association between NLR, PLR, NAR, PNI, SII or RA with 30-day mortality was found in ischemic stroke patients, this might because some other variables related to 30-day mortality of ischemic stroke patients were not included. The association between NAR and 30-day mortality of ischemic stroke patients showed a *P-*value of 0.055, this suggested that there might be association between NAR and 30-day mortality of ischemic stroke patients.

The strength in our study was that we focused on the prognostic values of LR, PLR, NAR, PNI, SII, and RA for 30-day mortality of different subtypes of stroke including ischemic stroke or hemorrhagic stroke. The finding might help identify potential reliable biomarkers in predicting those with high risk of 30-day mortality of different subtypes of stroke. There were several limitations in the current study. Firstly, this was a retrospective study from single-center, recall bias might exist. Secondly, due to the limitation of the database, some variables including the site or size of the hemorrhage or ischaemic stroke were unavailable, which might affect the results of our study. Thirdly, we analyzed the baseline data of inflammation biomarkers in ICU, and in the future, dynamic changes of the inflammation biomarkers during ICU stay will be analyzed to verify the results of our study. We will also conduct a study based on the samples from our hospital, and more important variables will be included.

## Conclusion

This study evaluated the predicitive values of NLR, PLR, NAR, PNI, SII, and RA for 30-day mortality of ischemic stroke or hemorrhagic stroke patients. We found that NLR, NAR and RA were potential prognostic biomarkers for predicting 30-day mortality in hemorrhagic stroke patients, which might help clinicians enhance risk stratification, design individual treatments, and determine follow-up schedules for hemorrhagic stroke patients.

## Data availability statement

Publicly available datasets were analyzed in this study. This data can be found in MIMIC-III database.

## Ethics statement

Requirement for individual patient consent was waived because the project did not impact clinical care and all protected health information was deidentified. As the samples were not from The Eighth Medical Center of PLA General Hospital, and this study was exempt from our Institutional Review Board approval.

## Author contributions

JZ and JF collected and analyzed the clinical data, reviewed the literature, and drafted the article. CL and FQ designed the study, supervised the initial drafting, and critically revised the article. All authors contributed to the article and approved the submitted version.

## References

[1] Campbell BCV Khatri P. Stroke. Lancet. (2020) 396:129–42. 10.1016/S0140-6736(20)31179-X 32653056

[2] Guzik A Bushnell C. Stroke Epidemiology and Risk Factor Management. Continuum. (2017) 23:15–39. 10.1212/CON.0000000000000416 28157742

[3] Abdu H Tadese F Seyoum G. Comparison of ischemic and hemorrhagic stroke in the medical ward of dessie referral hospital, northeast ethiopia: a retrospective study. Neurol Res Int. (2021) 2021:9996958. 10.1155/2021/9996958 34258063 PMC8257343

[4] Feigin VL Stark BA Johnson CO Roth GA Bisignano C Abady GG. Global, regional, and national burden of stroke and its risk factors, 1990–2019: a systematic analysis for the Global Burden of Disease Study 2019. Lancet Neurol. (2021) 20:795–820. 10.1016/S1474-4422(21)00252-0 34487721 PMC8443449

[5] Murray CJ Lopez AD. Measuring the global burden of disease. N Engl J Med. (2013) 369:448–57. 10.1056/NEJMra1201534 23902484

[6] Goyal M Menon BK van Zwam WH Dippel DW Mitchell PJ Demchuk AM. Endovascular thrombectomy after large-vessel ischaemic stroke: a meta-analysis of individual patient data from five randomised trials. Lancet. (2016) 387:1723–31. 10.1016/S0140-6736(16)00163-X 26898852

[7] Chen S Zeng L Hu Z. Progressing haemorrhagic stroke: categories, causes, mechanisms and managements. J Neurol. (2014) 261:2061–78. 10.1007/s00415-014-7291-1 24595959 PMC4221651

[8] Parikh NS Merkler AE Iadecola C. Inflammation, autoimmunity, infection, and stroke: epidemiology and lessons from therapeutic intervention. Stroke. (2020) 51:711–8. 10.1161/STROKEAHA.119.024157 32078460 PMC7041866

[9] Dong X Gao J Zhang CY Hayworth C Frank M Wang Z. Neutrophil membrane-derived nanovesicles alleviate inflammation to protect mouse brain injury from ischemic stroke. ACS Nano. (2019) 13:1272–83. 10.1021/acsnano.8b06572 30673266 PMC6424134

[10] Omar T Karakayali M Yesin M Alaydin HC Karabag Y Gümüşdag A. Monocyte to high-density lipoprotein cholesterol ratio is associated with the presence of carotid artery disease in acute ischemic stroke. Biomark Med. (2021) 15:489–95. 10.2217/bmm-2020-0705 33856263

[11] Aslan S Demir AR Demir Y Taşbulak Ö Altunova M Karakayali M. Usefulness of plateletcrit in the prediction of major adverse cardiac and cerebrovascular events in patients with carotid artery stenosis. Vascular. (2019) 27:479–86. 10.1177/1708538119847898 31027469

[12] Li W Hou M Ding Z Liu X Shao Y Li X. Prognostic value of neutrophil-to-lymphocyte ratio in stroke: a systematic review and meta-analysis. Front neurol. (2021) 12:686983. 10.3389/fneur.2021.686983 34630275 PMC8497704

[13] Yan YK Huang H Li DP Ai ZY Li X Sun Z. Prognostic value of the platelet-to-lymphocyte ratio for outcomes of stroke: a systematic review and meta-analysis. Eur Rev Med Pharmacol Sci. (2021) 25:6529–38. 10.26355/eurrev_202111_27095 34787855

[14] Chen Z Xie D Li Y Dai Z Xiang S Chen Z. Neutrophil albumin ratio is associated with all-cause mortality in stroke patients: a retrospective database study. Int J Gen Med. (2022) 15:1–9. 10.2147/IJGM.S323114 35018109 PMC8742575

[15] Liu Y Yang X Kadasah S Peng C. Clinical value of the prognostic nutrition index in the assessment of prognosis in critically Ill patients with stroke: a retrospective analysis. Comput Math Methods Med. (2022) 2022:4889920. 10.1155/2022/4889920 35586667 PMC9110188

[16] Ji Y Xu X Wu K Sun Y Wang H Guo Y. Prognosis of ischemic stroke patients undergoing endovascular thrombectomy is influenced by systemic inflammatory index through malignant brain edema. Clin Interv Aging. (2022) 17:1001–12. 10.2147/CIA.S365553 35814350 PMC9259057

[17] Zhao N Hu W Wu Z Wu X Li W Wang Y. The red blood cell distribution width-albumin ratio: a promising predictor of mortality in stroke patients. Int J Gen Med. (2021) 14:3737–47. 10.2147/IJGM.S322441 34326660 PMC8315287

[18] Johnson AE Pollard TJ Shen L Lehman LW Feng M Ghassemi M. MIMIC-III, a freely accessible critical care database. Scientific data. (2016) 3:160035. 10.1038/sdata.2016.35 27219127 PMC4878278

[19] Elkind MSV Boehme AK Smith CJ Meisel A Buckwalter MS. Infection as a stroke risk factor and determinant of outcome after stroke. Stroke. (2020) 51:3156–68. 10.1161/STROKEAHA.120.030429 32897811 PMC7530056

[20] Hermann DM Kleinschnitz C Gunzer M. Implications of polymorphonuclear neutrophils for ischemic stroke and intracerebral hemorrhage: predictive value, pathophysiological consequences and utility as therapeutic target. J Neuroimmunol. (2018) 321:138–43. 10.1016/j.jneuroim.2018.04.015 29729895

[21] Davidson S Coles M Thomas T Kollias G Ludewig B Turley S. Fibroblasts as immune regulators in infection, inflammation and cancer. Nat Rev Immunol. (2021) 21:704–17. 10.1038/s41577-021-00540-z 33911232

[22] Wanrooy BJ Wen SW Wong CH. Dynamic roles of neutrophils in post-stroke neuroinflammation. Immunol Cell Biol. (2021) 99:924–35. 10.1111/imcb.12463 33894069

[23] Xie W Li P. Visualizing regulatory lymphocytic responses to predict neurological outcome after stroke. CNS Neurosci Ther. (2021) 27:867–8. 10.1111/cns.13698 34156147 PMC8265945

[24] Drăgoescu AN Pădureanu V Stănculescu AD Chiu?u LC Tomescu P Geormăneanu C. Neutrophil to lymphocyte ratio (NLR)-a useful tool for the prognosis of sepsis in the ICU. Biomedicines. (2021) 10:75. 10.3390/biomedicines10010075 35052755 PMC8772781

[25] Lattanzi S Cagnetti C Provinciali L Silvestrini M. Neutrophil-to-lymphocyte ratio predicts the outcome of acute intracerebral hemorrhage. Stroke. (2016) 47:1654–7. 10.1161/STROKEAHA.116.013627 27165957

[26] Giede-Jeppe A Bobinger T Gerner ST Sembill JA Sprügel MI Beuscher VD. Neutrophil-to-lymphocyte ratio is an independent predictor for in-hospital mortality in spontaneous intracerebral hemorrhage. Cerebrovascular Dis. (2017) 44:26–34. 10.1159/000468996 28419988

[27] Han Z He X Peng S. Neutrophil count to albumin ratio as a prognostic indicator for HBV-associated decompensated cirrhosis. J Clin Lab Anal. (2021) 35:e23730. 10.1002/jcla.23730 33609049 PMC8059716

[28] Roche M Rondeau P Singh NR Tarnus E Bourdon E. The antioxidant properties of serum albumin. FEBS Lett. (2008) 582:1783–7. 10.1016/j.febslet.2008.04.057 18474236

[29] Akirov A Masri-Iraqi H Atamna A Shimon I. Corrigendum to 'low albumin levels are associated with mortality risk in hospitalized patients. Am J Med. (2020) 133:646. 10.1016/j.amjmed.2020.02.001 32127192

[30] Li J Imano H Yamagishi K Cui R Muraki I Umesawa M. Serum albumin and risks of stroke and its subtypes-the circulatory risk in communities study (CIRCS). Circ J. (2021) 85:385–92. 10.1253/circj.CJ-20-0384 33191391

[31] Zhao H Zhao Y Wu Z Cheng Y Zhao N. Red cell distribution width is associated with all-cause mortality in patients with acute stroke: a retrospective analysis of a large clinical database. J Int Med Res. (2021) 49:300060520980587. 10.1177/0300060520980587 33530799 PMC7871051

[32] Hou H Sun T Li C Li Y Guo Z Wang W. An overall and dose-response meta-analysis of red blood cell distribution width and CVD outcomes. Sci Rep. (2017) 7:43420. 10.1038/srep43420 28233844 PMC5324076

[33] Nakashima K Ohgami E Kato K Yoshitomi S Maruyama T Harada M. Prognostic significance of red cell distribution width in hospitalized older patients with heart failure or infection. Geriatr Gerontol Int. (2019) 19:988–92. 10.1111/ggi.13755 31397034

[34] Fan H Liu X Li S Liu P Song Y Wang H. High red blood cell distribution width levels could increase the risk of hemorrhagic transformation after intravenous thrombolysis in acute ischemic stroke patients. Aging. (2021) 13:20762–73. 10.18632/aging.203465 34449439 PMC8436933

[35] Petrone AB Eisenman RD Steele KN Mosmiller LT Urhie O Zdilla MJ. Temporal dynamics of peripheral neutrophil and lymphocytes following acute ischemic stroke. Neurol Sci. (2019) 40:1877–85. 10.1007/s10072-019-03919-y 31069585 PMC7898194

[36] Cai W Liu S Hu M Huang F Zhu Q Qiu W. Functional dynamics of neutrophils after ischemic stroke. Transl Stroke Res. (2020) 11:108–21. 10.1007/s12975-019-00694-y 30847778 PMC6993940

[37] Gill D Veltkamp R. Dynamics of T cell responses after stroke. Curr Opin Pharmacol. (2016) 26:26–32. 10.1016/j.coph.2015.09.009 26452204

[38] Paar M Rossmann C Nusshold C Wagner T Schlagenhauf A Leschnik B. Anticoagulant action of low, physiologic, and high albumin levels in whole blood. PLoS ONE. (2017) 12:e0182997. 10.1371/journal.pone.0182997 28800610 PMC5553770

[39] Purdon AD Rao AK. Interaction of albumin, arachidonic acid and prostanoids in platelets. Prostaglandins Leukot Essent Fatty Acids. (1989) 35:213–8. 10.1016/0952-3278(89)90004-5 2654961

[40] Li N Zhou H Tang Q. Red blood cell distribution width: a novel predictive indicator for cardiovascular and cerebrovascular diseases. Dis Markers. (2017) 2017:7089493. 10.1155/2017/7089493 29038615 PMC5606102

[41] Hemphill JC. 3rd, Bonovich DC, Besmertis L, Manley GT, Johnston SC: The ICH score: a simple, reliable grading scale for intracerebral hemorrhage. Stroke. (2001) 32:891–7. 10.1161/01.STR.32.4.891 11283388

[42] Huang LY Sun FR Yin JJ Ma YH Li HQ Zhong XL Yu JT Song JH. Associations of the neutrophil to lymphocyte ratio with intracranial artery stenosis and ischemic stroke. BMC Neurol. (2021) 21:56. 10.1186/s12883-021-02073-3 33546646 PMC7863476

[43] Yang Y Xie D Zhang Y. Increased platelet-to-lymphocyte ratio is an independent predictor of hemorrhagic transformation and in-hospital mortality among acute ischemic stroke with large-artery atherosclerosis patients. Int J Gen Med. (2021) 14:7545–55. 10.2147/IJGM.S329398 34754227 PMC8570380

[44] Xue J Huang W Chen X Li Q Cai Z Yu T. Neutrophil-to-lymphocyte ratio is a prognostic marker in acute ischemic stroke. J Stroke Cerebrovasc Dis. (2017) 26:650–7. 10.1016/j.jstrokecerebrovasdis.2016.11.010 27955949

[45] Bakogiannis C Sachse M Stamatelopoulos K Stellos K. Platelet-derived chemokines in inflammation and atherosclerosis. Cytokine. (2019) 122:154157. 10.1016/j.cyto.2017.09.013 29198385

[46] Kleinig TJ Vink R. Suppression of inflammation in ischemic and hemorrhagic stroke: therapeutic options. Curr Opin Neurol. (2009) 22:294–301. 10.1097/WCO.0b013e32832b4db3 19434798

[47] Yuan K Zhu S Wang H Chen J Zhang X Xu P. Association between malnutrition and long-term mortality in older adults with ischemic stroke. Clin Nutr. (2021) 40:2535–42. 10.1016/j.clnu.2021.04.018 33932800

